# DNA methylation and socioeconomic status in a Mexican-American birth cohort

**DOI:** 10.1186/s13148-018-0494-z

**Published:** 2018-05-08

**Authors:** Eric S. Coker, Robert Gunier, Karen Huen, Nina Holland, Brenda Eskenazi

**Affiliations:** 1 Center for Environmental Research and Children’s Health (CERCH), School of Public Health, University of California, Berkeley, CA USA; 2 Berkeley, USA; 3 Richmond, USA

**Keywords:** Diet, Epigenetics, Methylation, Repeat element, Social adversity, Socioeconomic status

## Abstract

**Background:**

Maternal social environmental stressors during pregnancy are associated with adverse birth and child developmental outcomes, and epigenetics has been proposed as a possible mechanism for such relationships.

**Methods:**

In a Mexican-American birth cohort of 241 maternal-infant pairs, cord blood samples were measured for repeat element DNA methylation (*LINE-1* and *Alu*). Linear mixed effects regression was used to model associations between indicators of the social environment (low household income and education, neighborhood-level characteristics) and repeat element methylation. Results from a dietary questionnaire were also used to assess the interaction between maternal diet quality and the social environment on markers of repeat element DNA methylation.

**Results:**

After adjusting for confounders, living in the most impoverished neighborhoods was associated with higher cord blood LINE-1 methylation (*β* = 0.78, 95%CI 0.06, 1.50, *p* = 0.03). No other neighborhood-, household-, or individual-level socioeconomic indicators were significantly associated with repeat element methylation. We observed a statistical trend showing that positive association between neighborhood poverty and LINE-1 methylation was strongest in cord blood of infants whose mothers reported better diet quality during pregnancy (*p*_interaction_ = 0.12).

**Conclusion:**

Our findings indicate a small yet unexpected positive association between neighborhood-level poverty during pregnancy and methylation of repetitive element DNA in infant cord blood and that this association is possibly modified by diet quality during pregnancy. However, our null findings for other adverse SES indicators do not provide strong evidence for an adverse association between early-life socioeconomic environment and repeat element DNA methylation in infants.

**Electronic supplementary material:**

The online version of this article (10.1186/s13148-018-0494-z) contains supplementary material, which is available to authorized users.

## Background

Social disadvantage in early-life can adversely affect child development as evidenced by the higher rates of preterm birth and low birth weight, asthma, and poorer cognitive function of children from economically impoverished families and neighborhoods [1–10]. While the precise mechanisms by which social disadvantage affects child health is not fully understood, it has been hypothesized that maternal exposure to social stressors during pregnancy, such as poverty, may alter the offspring’s epigenome [11, 12].

DNA methylation, which is the attachment of methyl groups primarily at CpG sites, is an example of an epigenetic modification that can regulate gene expression [13, 14], with methylation of repetitive elements being one commonly studied epigenetic marker. Repetitive elements make up approximately half of the human genome and include retrotransposable elements like long and short interspersed nuclear elements (LINEs and SINEs, respectively). Of the LINEs and SINES, the LINE-1 and Alu elements are the most abundant, together representing nearly a third of the human genome [15]. Although previously referred to as markers of global methylation, recent studies have demonstrated that LINE-1 and Alu methylation do not correlate well with global genomic methylation content [16, 17], are not correlated with each other [18, 19], and each represents distinct and important components of the epigenome [17, 20].

In the present study, we focus on perinatal LINE-1 and Alu methylation related to the maternal socioeconomic environment because previous epidemiological studies suggest associations with social and environmental stressors on LINE-1 and Alu methylation in both children and in adults. Both human and animal studies indicate hypomethylation or high copy numbers of LINE-1 or SINE elements in relation to stress or psychiatric disorders [21–25]. DNA methylation of LINE-1 and Alu elements have also been associated with environmental pollutant exposures (both hypomethylation and hypermethylation) [26–31], diet and nutrition [32–34], and other lifestyle factors [31, 35]. These relationships may have biological relevance since hypomethylation of repetitive elements may affect genomic instability and later disease states [36, 37].

Few studies have examined the relationships between early-life socioeconomic disadvantage and methylation in newborn blood or placental tissue. A birth cohort study conducted in New York [38] found no association between maternal education during pregnancy and global DNA methylation measured by immunoassay in infant cord blood, while another study in Rhode Island found maternal socioeconomic disadvantage, including lower education level, to be related to placental hypomethylation of the *HSD11B2* gene (that may affect maternal cortisol exposure in the infant) [39]. Meanwhile, a birth cohort study from North Carolina found that lower maternal education and household income were associated with hypomethylation of *IGF2* and *H19* imprinted genes but there was no association with other genes (*MEG3* and *NNAT*) [40]. In a separate analysis in this same North Carolina cohort, authors observed an opposite and significant association with neighborhood socioeconomic status (SES) for *MEG3* (hypermethylation) [41]. Finally, a birth cohort study from China [42] measured DNA methylation at several hundred CpG sites and found that lower maternal SES was associated with older epigenetic age using a novel ‘epigenetic clock’ method. However, repeat element methylation in infants has yet to be examined in relation to socioeconomic status.

Maternal nutrition may be an important co-exposure to consider in the context of socioeconomic disadvantage because neighborhood food environments and poverty level [43–45], and maternal socioeconomic status and nutrition [46–48] have been associated with each other. In addition, prenatal nutrition is associated with changes in DNA methylation [32, 33, 49, 50]. For example, prospective studies have found that supplementation with methyl donors during pregnancy, such as folate, was associated with hypermethylation of LINE-1elements in cord and maternal blood [34, 49, 51]. In a Dutch population, epigenetic changes (hyper- or hypo methylation depending on the gene) have been observed with prenatal famine exposure in a time of great social stress (World War II) and these changes have been retained into adulthood along with modifications of the imprinted genes that were preserved for several generations of offspring [52, 53]. Thus, it appears plausible that good maternal diet during pregnancy may protect against the adverse effects of social adversity on the newborn epigenome.

In our study, we used a multi-level analysis to investigate associations between indicators of maternal socioeconomic status and DNA methylation of LINE-1 and Alu repeat elements in cord blood of infants who participated in the Center for Health Assessment of Mothers and Children of Salinas (CHAMACOS), a birth cohort study of predominantly economically disadvantaged Mexican-American farmworker families. We hypothesized that maternal socioeconomic status (SES) at the individual, household, and neighborhood level will influence the newborn epigenome as measured by LINE-1 and Alu repeat elements and that maternal diet quality will modify this association.

## Methods

### Study population

Pregnant women were recruited from six community health clinics in the Salinas Valley, California (1999–2000) and were eligible for the CHAMACOS study if they were at least 18 years of age, less than 20 weeks gestation, spoke Spanish or English, were eligible for low income health insurance, and were planning to deliver at the local public hospital. There were 601 women enrolled of whom 526 delivered a live singleton infant. Cord blood samples were collected at the time of delivery, of which 241 had sufficient DNA to analyze methylation of LINE-1 and Alu repeats and cell composition estimates. There were no significant differences in sociodemographic characteristics or important health behaviors (e.g., smoking) between the mothers of children who were included and those who were not included in these analyses (data not shown).

### Socioeconomic disadvantage and maternal diet during pregnancy

Women were interviewed to collect individual- and household-level indicators of SES and other demographic information (e.g., age and race). Maternal interviews occurred twice during pregnancy at ~ 13 and ~ 26 weeks gestation, and all interviews were administered in English or Spanish by trained bilingual interviewers.

#### Individual- and household-level variables

We obtained information on maternal education and household income, and the number of people living in the home at the first interview. Mother’s education was constructed into three categories (≤ 6th grade, 7–12th grade, and ≥ high school), while household poverty income ratio variables were classified into quartiles for analysis. Household poverty income ratio was computed by combining information on the reported household income and the number of people supported by that income and then dividing these values by the year 2000 US Census poverty threshold values for the number of people living in the household [54]. Hence, a poverty income ratio < 1 entails that the household is below the poverty line and conversely a poverty income ratio ≥ 1 entails that the household is above the poverty line.

#### Neighborhood-level variables

Census tract data were obtained for the year 2000 from the US Census Bureau. We examined sociodemographic measures for the census tract the woman lived in including: percent of homes below the poverty line, median household income, and percent of people that completed high school within census tracts. Each neighborhood SES variable was categorized into quartiles for analysis.

#### Dietary intake during pregnancy

Information regarding dietary and nutritional supplement intake were ascertained at the second prenatal interview (~ 26 weeks gestation) [54]. Briefly, we used a validated 72-item food-frequency questionnaire (FFQ) that was developed for Spanish-speaking populations [55, 56]. The FFQ collected information on typical food intake such as frequency and portion sizes for specific food items. We converted food and vitamin supplement intake into average daily energy and nutrient intake as set out by United States Department of Agriculture Nutrient Database for Standard Reference [57]. With data from the FFQ, we determined maternal diet quality, using the Diet Quality Index for Pregnancy (DQI-P) [58]. The original DQI-P comprises eight different components of dietary intake; however, we had data for seven of these components (we were not able to include the component for the number of meals and snacks per day). The seven components assessed the adequacy of intake grains, fruits, and vegetables; the intake of fat as a proportion of all energy intake; and the adequacy of specific nutrient intake of folate, iron, and calcium. A higher DQI-P score is indicative of better diet quality while a lower score is indicative of poorer diet quality. For greater details on how these data were collected and DQI-*P* values were derived, see Harley et al. 2006 [54].

### DNA methylation in cord blood

Umbilical cord blood samples were collected at the delivery room. Samples were centrifuged and divided into serum and clot and stored at − 80 °C [59]. DNA were isolated from clots with a QIAamp Blood DNA Maxi kit (Qiagen, Inc., Santa Clarita, CA, USA) (further details described in Holland et al. 2006) [59]. For bisulfite conversion of 500 ng of DNA, we used EpiTect Bisulfite Conversion Kits (Qiagen, Germantown, MD, USA). Bisulfite converted samples were then eluted into 20 μL Elution buffer. To confirm complete bisulfite conversion, we calculated the proportion of cytosine at the first non-CpG site, considering proportions over 7% as an indicator of incomplete bisulfite conversion. Cytosine proportions for the non-CpG site in individual samples reflective of incomplete bisulfite conversion ranged from 0.1 to 4.2% (mean = 1.5%), confirming excellent conversion efficiency for the majority of samples (99%) [26]. Pyrosequencing of PCR-amplified and bisulfite-treated DNA samples was used to determine LINE-1 and Alu methylation levels, using the Pyromark Q96MD System (Qiagen). Methylation of LINE-1 and Alu (%5-mC [%5-methylated cytosine]) was calculated at each of the four known CpG sites and for each triplicate analysis of each participant sample using Pyro Q-CpG Software (Qiagen) [60, 61]. Details of bisulfite pyrosequencing of Alu and LINE-1 methylation have been described by previous studies [60, 62].

A combination of QA/QC approaches was used to reduce technical and batch sources of variability. We included a no template control (NTC) in addition to unmethylated, partially methylated, and completely methylated genomic DNA. Intraplate repeats of samples were also randomly distributed across plates. We ran all sample plates on the same day in order to limit batch variability. All coefficients of variation for repeat measures and intraplate coefficients of variation were in acceptable ranges (≤ 5%) [26], and all isolated DNA samples had 260/280 ratios > 1.6.

### Covariates

Covariates were selected for fully adjusted models based on the literature [26, 51, 63–65] and a directed acyclic graph (DAG) (Additional file 1: Figure S1). For all models, confounders included maternal age at delivery, the number of years living in the USA (≤ 1 year, 2–5 years, 6–10 years, 11–24 years, and entire life), maternal smoking during pregnancy, maternal diet quality during pregnancy (DQI-P), and maternal urinary phthalate concentrations of monobenzyl phthalate (MBzP) during pregnancy. Maternal age was obtained during the baseline interview as was tobacco smoking status during pregnancy. In a previous work, we showed that prenatal exposure to urinary MBzP (a phthalate metabolite) was inversely associated with LINE-1 methylation [26] and that indicators of maternal SES were moderately to highly correlated with urinary MBzP [66]. Maternal urinary MBzP concentrations were determined for pregnancy by collecting maternal urine at each prenatal interview and measured by on-line solid phase extraction coupled with isotope dilution-high-performance liquid chromatography-electrospray ionization-tandem mass spectrometry at the Center for Disease Control and Prevention [67]. The two measurements were averaged to derive an overall pregnancy average concentration. We used log-transformed values of MBzP in the regression analyses. Cell composition was controlled for in sensitivity analyses. Cell composition estimation was performed using a recently validated reference database of nucleated cord blood cell types [68, 69]. Cell types included CD8T, CD4T, Natural killer, B lymphocytes, Monocytes, Granulocytes, and nucleated red blood cells.

### Statistical analysis

Summary statistics were computed for all study covariates, exposures of interest, and basic study population characteristics. Linear mixed effects regression was used to detect associations between LINE-1 and Alu DNA methylation in cord blood and indicators of the social environment at the individual, household, and neighborhood levels, and for maternal diet quality during pregnancy (DQI-P). Continuous SES indicator variables (percent below poverty, median household income, percent with a high school education, and poverty income ratio) were modeled in separate analyses as continuous and in quartiles (to account for potential non-linearity). We then explored whether SES associations with repeat element methylation were modified by prenatal maternal diet quality. Consistent with our previous work [26, 27], we ran mixed effects models with a random effect for repeat analyses (triplicate) of DNA methylation to account for correlation within subject and a separate random effect for methylation sites (four CpG sites analyzed for both LINE-1 and Alu assays) to account for site-specific correlation. In our main analyses looking at socioeconomic indicators and diet quality, we implemented three separate mixed effects models. In model 1, we only controlled for random effects to estimate crude associations between exposures of interest and methylation. In model 2 we adjusted for maternal factors including smoking during pregnancy, age at delivery, MBzP urinary concentrations, and diet quality. Model 3 was a sensitivity analysis, where we adjusted for cell type estimates since methylation is likely to be dependent on cell type. Finally, to test for interaction between diet quality and measures of socioeconomic disadvantage, we applied interaction terms between each categorical socioeconomic indicator variable and DQI-P (diet quality, modeled continuous), adjusting for all other covariates in model 2. For illustrative purposes and to display interaction effects, we consider stratified analyses by DQI-P quintiles. All summary statistics and regression models were analyzed with R statistical software (version 3.2.4) using the *lmer* function (lmerTest, version 2.0–32) for linear mixed effects regression models. A *p* value < 0.05 and < 0.1 were considered statistically significant and marginally significant, respectively, for main effects. Since there are simultaneous tests when assessing for interaction between a categorical variable (e.g., quantiles) and another variable, we applied a partial sum of squares *F* test (*anova* command in R) in order to report on *p* values for the diet quality (significance-level at *p*_interaction_ < 0.05). In addition to *p* values, we also qualitatively assessed the heterogeneity in effect sizes when stratifying by quintiles of diet quality. In order to take advantage of the complete data on the study outcomes, missing values on the covariates included in the regression models were imputed using factorial analysis for mixed data on both continuous and categorical variables (R package: *missMDA*). The percent missing for the three imputed covariates were as follows: < 1% (*N* = 1) on maternal MBzP concentration, < 1% (*N* = 1) on maternal age, and 5% (*N* = 13) on DQI-P.

## Results

Maternal and infant characteristics included in our analysis are presented in Table 1. Newborns were evenly split between males (49.8%) and females (50.2%) with mean gestation length and birth weight of 39 weeks and 3467 g, respectively. Most of the mothers were Latina (96%) with an average maternal age at delivery of 26 years. Mean pre-pregnancy BMI was 26.5 kg/m^2^, with the majority of mothers classified as either overweight (37%) or obese (20%) prior to pregnancy.

**Table 1:** Summary statistics of participating infants and mothers, CHAMACOS (*n* = 241 mother-infant dyads)

	All
Infant	
Preterm birth, *N* (%)	
Yes	13 (5.4)
No	228 (94.6)
Low birth weight, *N* (%)	
Yes	7 (2.9)
No	234 (97.1)
Mother	
Age at pregnancy, years (M ± SD)	25.8 ± 5.3
Pre-pregnancy body mass index, kg/m^2^ (M ± SD)	26.5 ± 5.0
Race/ethnicity, *N* (%)	
Latino	232 (96.2)
White	5 (2.1)
Other	4 (1.7)
Educational attainment, *N* (%)	
< 6th grade	98 (40.7)
7–12 grade	92 (38.2)
≥ High school	51 (21.1)
Country of birth, *N* (%)	
U.S.	32 (13.3)
Mexico	205 (85.1)
Central America/other	4 (1.7)
Years spent living in the U.S., *N* (%)	
< =1 year	61 (25.3)
2–5 years	70 (29.0)
6–10 years	48 (19.9)
11–24 years	34 (14.1)
Entire life (18–32 years)	28 (11.6)
Diet quality index during pregnancy (M ± SD)	44.90 ± 9.66
Urinary MBzP^a^, μg/L (IQR^b^)	9.5 (4.7, 17.8)

^a^Median urinary concentration, limit of detection is 0.3 μg/L

^b^*IQR* interquartile range

Individual-level socioeconomic disadvantage (Table 1) and household-level socioeconomic disadvantage (Table 2) was severe in the study population. A majority of mothers have less than a high school degree (79%) and live in households with a poverty income ratio < 1 (70%). Neighborhood socioeconomic disadvantage was similarly high, with a tract-level median of $34, 211 for median household income and median percentages of 24% for household poverty and 73% without a high school education. Correlations between maternal- or household- and neighborhood-level socioeconomic class indicators were generally weak although mostly in the anticipated directions (Additional file 1: Figure S2). Of all the study covariates included in adjusted regression models, MBzP and the number of years living in the USA exhibited the strongest correlations with the SES variables of interest for this study (*r* = − 0.27 to 0.30). Maternal smoking was only weakly correlated with household-level poverty income ratio (*r* = 0.10) and income (*r* = 0.06), moderately correlated with the number of years living in the USA (*r* = 0.27), and moderately correlated with neighborhood SES (range *r* = − 0.23, 0.22).

**Table 2:** Summary statistics of SES at the household and census tract level, CHAMACOS (*n* = 241)

SES indicators	
Household	Median (IQR)
Income, monthly ($)	375.2 (281.4, 562.8)
Poverty income ratio	0.98 (0.65, 1.20)
Neighborhood (census tracts)	Median (IQR)
Household income, yearly ($)	34211 (31910, 41354)
% of household below poverty	23.50 (19.38, 27.54)
% of people with no high school diploma	72.80 (50.10, 75.34)

### DNA methylation: LINE-1 and Alu

Average LINE-1 methylation was 78.9 (%5-mC) (95%CI 78.7, 79.1). As much as 11% (chi-squared test *p* value < 0.05) of the overall variance in LINE-1 methylation was explained by differences between CpG sites (suggesting significant level 2 CpG site effects). LINE-1 methylation levels were also slightly higher in boys compared to girls (*p* = 0.03), marginally lower in low birth weight infants compared to normal weight infants (*p* = 0.06), but there was no association between preterm infants and LINE-1 methylation. The summary statistics for LINE-1 methylation, stratified by quantiles of the SES indicators, are presented in Table 3. Average Alu methylation was 25.3 (%5-mC) (95%CI 25.1, 25.5) and clustering of methylation levels by CpG site. There were no significant differences in Alu methylation levels between boys and girls or by preterm or low birth weight of the infant. Confirming our previous work, maternal urinary MBzP consistently resulted in a statistically significant linear negative association with LINE-1 methylation (model 2). Interestingly, fewer years spent living in the USA (≤ 1 year and 2–5 years) consistently had significantly higher LINE-1 methylation compared to those who lived their entire life in the USA.

**Table 3:** Mean LINE-1% methylation overall and stratified by SES indicator categories and years living in the USA

SES indicators	LINE-1% mean methylation (95% CI)
Overall	78.9 (78.7, 79.1)
Maternal-level	
Educational attainment	
< 6th grade	78.8 (78.5, 79.1)
7th–12th grade	79.2 (78.8, 79.4)
≥ High school	78.7 (78.3, 79.2)
Household-level	
Monthly income quartiles	
1st quartile ($37–$225)	78.7 (78.3, 79.2)
2nd quartile ($281–$375)	79.1 (78.8, 79.4)
3rd quartile ($438–$563)	78.8 (78.4, 79.2)
4th quartile ($583–$1750)	78.7 (78.1, 79.2)
Poverty income ratio quartiles	
1st quartile (0.13–0.65)	78.8 (78.4, 79.2)
2nd quartile (0.71–0.98)	79.1 (78.8, 79.5)
3rd quartile (1.01–1.21)	78.9 (78.8, 79.3)
4th quartile (1.30–2.40)	78.7 (78.2, 79.1)
Neighborhood (census tract)-level	
Median household income quartiles	
1st quartile ($24,896–$31910)	78.9 (78.6, 79.2)
2nd quartile ($31,989–$34593)	78.7 (78.2, 79.2)
3rd quartile ($34,848–$40856)	78.9 (78.5, 79.4)
4th quartile ($41,354–$77272)	79.0 (78.6, 79.4)
Percent below poverty quartiles	
1st quartile (2.8–18.2)	78.7 (78.3, 79.1)
2nd quartile (19.0–22.3)	79.0 (78.6, 79.4)
3rd quartile (23.5–27.5)	78.7 (78.4, 79.0)
4th quartile (27.7–34.2)	79.8 (79.1, 80.4)
Percent without a highschool education quartiles	
1st quartile (13.7–50.1)	78.9 (78.5, 79.3)
2nd quartile (51.4–71.4)	79.1 (78.7, 79.5)
3rd quartile (72.5–75.3)	78.6 (78.2, 79.0)
4th quartile (78.7–87.0)	78.9 (78.6, 79.3)
Years living in the USA (“acculturation”)	
< =1 year	79.2 (78.6, 79.7)
2–5 years	79.3 (78.9, 79.8)
6–10 years	78.8 (78.4, 79.8)
11–24 years	78.8 (78.4, 79.2)
Entire life (18–32 years)	78.4 (77.8, 78.9)

### Associations of SES and DNA methylation

The summary statistics for LINE-1 methylation, stratified by the different SES categories, are presented in Additional file 1: Table S1. There were no significant differences in LINE-1 methylation by neighborhood income levels or by neighborhood educational attainment, in both crude (model 1) and adjusted (model 2) analyses (Table 4). Living in the highest poverty neighborhood quartile was significantly associated with higher LINE-1 methylation compared to living in the lowest poverty neighborhood quartile (adjusted β = 0.78, 95%CI: 0.06, 1.50, *p* = 0.03). There was a lack of a linear trend (*p* = 0.51) for increasing neighborhood poverty quartiles and methylation. We found no statistically significant differences in LINE-1 methylation by individual- or household-level SES (Table 4) in crude or adjusted models. Although after adjustment, the second household income quartile and second poverty income ratio quartile were marginally associated with higher methylation compared to the highest SES quartile categories (*p*-value = 0.05 and 0.06, respectively). After controlling for estimated cell type proportions (Model 3, Table 4), the neighborhood-level poverty association with LINE-1 methylation strengthened slightly (*β* = 0.88, *p* = 0.02), but the household-level SES variables attenuated substantially and were no longer marginally associated after controlling for cell type proportions. No significant associations of socioeconomic indicators were observed with Alu methylation (Additional file 1: Table S1).

**Table 4:** Results from linear regression mixed effects (LMER) models of crude and adjusted associations between maternal, household, and neighborhood indicators of SES and diet quality index and LINE-1 DNA methylation

Socioeconomic status indicators	Model 1^a^		Model 2^b^		Model 3^c^	
	*β* (95% CI)	*p* value	aβ (95% CI)	*p* value	aβ (95% CI)	*p* value
Household income						
1st quartile ($37–$225)	0.05 (−0.59, 0.70)	0.88	0.08 (−0.59, 0.75)	0.82	−0.18 (−0.87, 0.52)	0.62
2nd quartile ($281–$375)	0.46 (− 0.13, 1.04)	0.12	0.60 (− 0.01, 1.21)	0.05	0.18 (− 0.48, 0.83)	0.60
3rd quartile ($438–$563)	0.14 (−0.52, 0.80)	0.68	0.20 (−0.46, 0.86)	0.56	−0.07 (− 0.75, 0.60)	0.84
4th quartile ($583–$1750)	Reference		Reference		Reference	
Household poverty income ratio						
1st quartile (0.13–0.65)	0.07 (−0.49, 0.63)	0.80	0.12 (−0.45, 0.69)	0.68	−0.18 (− 0.78, 0.41)	0.54
2nd quartile (0.71–0.98)	0.08 (− 0.12, 1.01)	0.09	0.53 (− 0.03, 1.08)	0.07	0.15 (− 0.45, 0.75)	0.62
3rd quartile (1.01–1.21)	0.45 (− 0.42, 0.82)	0.56	0.21 (− 0.43, 0.85)	0.52	− 0.13 (− 0.78, 0.53)	0.71
4th quartile (1.30–2.40)	Reference		Reference		Reference	
Maternal education						
< = 6th grade	0.04 (−0.46, 0.53)	0.89	−0.09 (− 0.65, 0.47)	0.75	− 0.03 (− 0.60, 0.54)	0.92
7–12th grade	0.39 (− 0.12, 0.90)	0.13	0.33 (− 0.20, 0.86)	0.22	0.35 (− 0.18, 0.89)	0.20
> =Highschool	Reference		Reference			
% Households below poverty (CT)						
1st quartile (2.8–18.2)	Reference		Reference		Reference	
2nd quartile (19.0–22.3)	0.26 (−0.27, 0.80)	0.34	0.24 (−0.31, 0.78)	0.39	0.31 (−0.26, 0.85)	0.30
3rd quartile (23.5–27.5)	− 0.01 (− 0.49, 0.47)	0.97	−0.11 (− 0.61, 0.39)	0.68	−0.04 (− 0.47, 0.54)	0.89
4th quartile (27.7–34.2)	1.03 (0.33, 1.73)	0.004	0.78 (0.06, 1.50)	0.03	0.88 (0.14, 1.64)	0.02
Median household income (CT)						
1st quartile ($24,896–$31910)	−0.05 (− 0.53, 0.43)	0.84	− 0.03 (− 0.58, 0.53)	0.93	−0.02 (− 0.58, 0.53)	0.94
2nd quartile ($31,989–$34593)	−0.26 (− 0.84, 0.32)	0.39	−0.40 (− 0.93, 0.13)	0.14	−0.48 (−1.01, 0.05)	0.08
3rd quartile ($34,848–$40856)	0.02 (−0.61, 0.57)	0.94	−0.12 (− 0.66, 0.42)	0.66	− 0.01 (− 0.55, 0.53)	0.98
4th quartile ($41,354–$77272)	Reference		Reference		Reference	
% No highschool education (CT)						
1st quartile (13.7–50.1)	Reference		Reference		Reference	
2nd quartile (51.4–71.4)	0.16 (−0.38, 0.70)	0.55	0.07 (−0.49, 0.63)	0.81	0.08 (−0.48, 0.64)	0.77
3rd quartile (72.5–75.3)	−0.33 (− 0.84, 0.19)	0.21	−0.30 (− 0.84, 0.24)	0.28	−0.33(− 0.87, 0.22)	0.24
4th Quartile (78.7–87.0)	−0.02 (− 0.50, 0.54)	0.94	−0.10 (− 0.66, 0.46)	0.72	0.04 (− 0.53, 0.60)	0.90
Diet quality index	0.020 (−0.008, 0.032)	0.24	0.13 (−0.06, 0.32)^d^	0.19	0.08 (−0.12, 0.27)^d^	0.44

^a^Model 1: random effect for position and individual only

^b^Model 2: random effect for position and individual, maternal smoking during pregnancy, maternal age, diet quality during pregnancy, years living in the USA for the mother, and prenatal MBzP exposure

^c^Model 3: random effect for position and individual, maternal smoking during pregnancy, maternal age, diet quality during pregnancy, years living in the USA for the mother, prenatal MBzP exposure, and cell estimate proportions

^d^Neighborhood poverty included as a covariate due to evidence of confounding by neighborhood poverty. Diet quality index was *Z* standardized so that continuous variables were on similar scales

### Diet quality during pregnancy, SES, and LINE-1 methylation

**Fig. 1:**
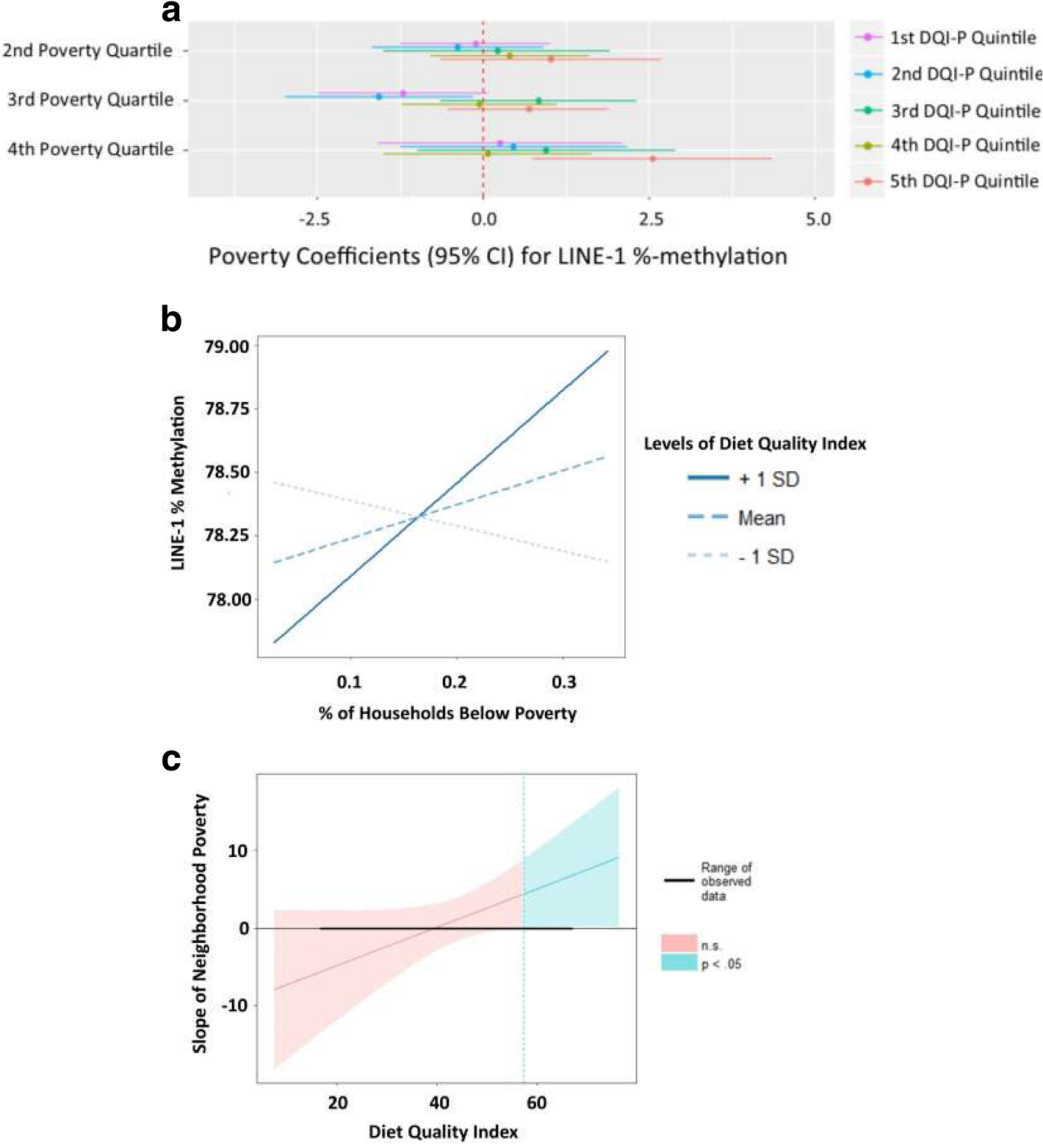
Interaction between neighborhood poverty and maternal diet quality during pregnancy (DQI-P). **a** Displays the regression coefficients stratified by different quintiles of diet quality index (DQI-P). Regression coefficients are interpreted as the adjusted difference in LINE-1%-methylation between a quartile of neighborhood poverty and the lowest neighborhood poverty quartile (reference). Each different color corresponds to a different stratum of DQI-P as indicated by the legend (e.g., purple is the first quintile of DQI-P and orange is the fifth quintile of DQI-P). **b** Displays the slopes for neighborhood poverty (*x* axis) on LINE-1%-methylation (*y* axis) at different levels of DQI-P (e.g., dark blue line is the slope for observations at + 1 standard deviation for DQI-P and the light gray line is the slope for observations at − 1 standard deviation for DQI-P). **c** Displays the simple slopes for neighborhood poverty (*y* axis) across different levels of DQI-P (*x* axis). The pink portion of the confidence interval indicates no statistical significance (*p* > 0.05) between poverty and LINE-1 methylation and the blue-green portion of the confidence interval indicates statistical significance (*p* < 0.05) between poverty and LINE-1 methylation. The vertical dotted line indicates the point at which the relationship between poverty and LINE-1 methylation becomes statistically significant

Diet quality index during pregnancy (DQI-P) was positively, albeit non-significantly, associated with LINE-1 methylation in crude and adjusted models (*p*_adjusted_ = 0.19) (Table 4). Including an interaction term between neighborhood poverty quartiles and DQI-P indicated a statistical trend towards interaction (*p*_interaction_ = 0.12). In Fig. 1a, we present the adjusted regression coefficients for each poverty quartiles on LINE-1 methylation when the regression analyses are stratified by DQI-P quintiles. We observed that in the highest quintile of DQI-P (best diet quality), the adjusted mean LINE-1 methylation was higher by 2.6% in the highest quartile of neighborhood poverty compared with the lowest poverty quartile. Conversely, in the lower DQI-P quintiles (poorer diet quality), there was either no differences in LINE-1 methylation or lower methylation with increasing neighborhood poverty quartiles compared to the lowest poverty quartile. When neighborhood poverty is modeled as a continuous variable along with an interaction term with DQI-P, this statistical trend strengthened (*p*_interaction_ = 0.06). As shown in Fig. 1b, at very low levels of maternal diet quality, there is a negative exposure-response relationship between neighborhood poverty and LINE-1 methylation, and conversely, at high levels of maternal diet quality, there is a positive exposure-response relationship between neighborhood poverty and LINE-1 methylation. In Fig. 1c, the relationship between neighborhood poverty and LINE-1 methylation is significant only at very high levels of maternal diet quality. There was no evidence for interaction between other indicators of SES and diet quality on LINE-1 methylation (*p*_interaction_ > 0.2 in all cases). Finally, we did not observe any differences in Alu methylation by diet (Additional file 1: Table S2) or by combination of diet and SES indicators (*p*_interaction_ > 0.2 in all cases).

## Discussion

In the CHAMACOS cohort, we observed a small but statistically significant association between the prenatal socioeconomic environment and DNA methylation of LINE-1 repeat elements in infant cord blood. Specifically, only higher neighborhood poverty was significantly associated with LINE-1 hypermethylation and this association was possibly moderated by the quality of maternal diet during pregnancy.

As already discussed in the introduction, previous studies relating to epigenetic markers with maternal SES or neighborhood SES indicators during pregnancy vary by methodology, design, and findings. For instance, a cohort study in NY that examined the relationship between maternal SES and global DNA methylation in infant cord blood found no associations [38]. Our findings also show a null association between maternal education and LINE-1 methylation but a positive relationship with neighborhood poverty. The NY study, however, did not consider neighborhood SES indicators. Furthermore, they used a measure of global methylation measured by immunoassay while our pyrosequencing analysis presented here focused on repetitive element methylation that does not necessarily correlate with global methylation.

Prenatal neighborhood-level poverty and individual-level poverty have each been associated with adverse developmental outcomes such as low birth weight and preterm birth [70]. In our study, we only observed an epigenetic association with neighborhood poverty. The precise mechanisms of neighborhood effects on health or epigenetic changes are not clear. Such neighborhood effects possibly result from the sustained and combined exposures to multiple adverse social and physical individual-level risk factors that may be experienced by individuals living in impoverished neighborhoods. The multiple social and physical risk factors can include low access to healthy foods [71] and health care, mental stress associated with violence and social isolation, higher exposure to environmental pollutants, and built environments that promote unhealthy behaviors [72]. Thus, one possible explanation behind our observing only neighborhood effects, rather than individual-level effects in our study, could be related to this concept of sustained and combined stressors tied to neighborhood SES, while a single parameter of individual socioeconomic adversity is unable to capture this accumulation of combined social, physical, and economic stressors. Additionally, our null findings for maternal-level and household-level socioeconomic indicators may be related to a relative homogeneity in low SES among the CHAMACOS participants.

Since neighborhood poverty has typically been associated with greater exposure to maternal stress as well as adverse developmental outcomes in previous studies, it may appear counterintuitive that the children of CHAMACOS mothers living in higher poverty neighborhoods during pregnancy are observed to have higher LINE-1 methylation in cord blood, relative to lower poverty neighborhoods. Unexpected observation of protective social adversity effects in utero is not unfounded however. The prenatal LINE-1 hypermethylation observed in our study could reflect an adaptive response to maternal stress cues that is intended to influence the development of a phenotype that is adapted to coping with a similarly stressful environment later in life [73]. In other words, hypermethylation of LINE-1 may be a response to stress that could make the individual more resilient to similar stressful events in the future. For instance, the timing of elevated maternal anxiety during pregnancy and related prenatal exposure to higher levels of cortisol in utero has been shown to be protective of mental health and neurodevelopment in infancy [74], and newborn DNA methylation has been linked with infant cortisol response (glucocorticoid receptor gene methylation) [75] and infant neurodevelopment (LINE-1 methylation) [76]. A potential adaptive phenomenon with LINE-1 hypermethylation has also been reported in a military service personnel with post-traumatic stress disorder [21].

Further, a positive association between higher neighborhood poverty and LINE-1 methylation was primarily seen in the cord blood of infants from CHAMACOS mothers who had the highest quality dietary patterns during pregnancy (Fig. 1a). This finding of the potentially moderating epigenetic effects between neighborhood poverty and maternal diet quality lends some support to the hypothesis that maternal diet may supply “epi-nutrients” that influence epigenetic effects from exposure to stressors in utero [77]. In addition, other studies suggest that neighborhood poverty and nutrition, and their health effects, may be closely linked. Neighborhood-level disadvantage has been associated with poorer diet quality [78], poorer nutritional status [79], and higher hair cortisol levels (an indicator of chronic stress), while better diet among those with lower SES has been shown to be protective against inflammatory [80] and poor mental health outcomes [81, 82]. Future studies should measure maternal diet quality or nutritional status when considering neighborhood SES or maternal stress effects on infant epigenetics in order to assess the potential moderating role of maternal diet.

While no human studies have used a diet quality index to investigate associations with epigenetic changes, some researchers have investigated associations of specific nutrients as well as eating disorders during pregnancy in relation to repeat element methylation. One human study [65] found that choline intake during pregnancy was significantly and inversely related to cord blood LINE-1 methylation in boys but positively associated with LINE-1 methylation in girls (albeit non-significant). Another human study reported that folate supplementation was inversely related with cord blood LINE-1 methylation [51]. A recent study [83] found that maternal eating disorders (restrictive, purging, binge eating, binge-purge) during pregnancy were associated with hypomethylation in infant cord blood, possibly related to lower calorie intake. Our finding of a positive association (albeit statistically insignificant) between better diet quality during pregnancy and repeat element methylation also supports the role of maternal diet as a determinant of infant methylation levels.

Although the consideration of SES at multiple levels (individual, household, and neighborhood) is an important strength of our study because each level may act independent of one another [84], it does not implicate any specific environmental stressors related to area poverty. In addition to the socioeconomic indicators we considered, other factors potentially may contribute to differential LINE-1 and Alu methylation such as prenatal exposures to persistent organic pollutants [27] and other chemicals [85], or other relevant biologic measures (e.g., BMI or cortisol levels [23]). Some of these effects have been assessed in previously published CHAMACOS studies [26, 27] and do not appear to be confounders in the current analyses. Although the homogeneity of the CHAMACOS population in the Salinas Valley, California is another potential limitation of our study, it is also a strength in that there is less likely to be uncontrolled confounding due to other factors. To make our findings more generalizable, additional studies in other ethnic and SES cohorts are warranted. It will be important to also further explore potential influence of maternal nutrition on associations between the socioeconomic status and DNA methylation.

## Conclusions

We observed a modest but statistically significant association between neighborhood-level poverty and LINE-1 repeat element methylation in newborns, and the direction of this relationship appears to be modified by maternal diet during pregnancy. This finding implies that maternal diet may have a moderating effect on the association between neighborhood poverty and repeat element DNA methylation and that future epigenetic studies investigating the prenatal effects of social class should consider maternal diet as a potential moderator of neighborhood socioeconomic effects on epigenetics.

## Additional files

Additional file 1: **Figure S1.** Minimal sufficient adjustment for measuring the direct effect of SES on LINE-1 DNA methylation: diet quality index, maternal urinary phthalate concentration, maternal age, maternal smoking during pregnancy, and the number of years living in the U.S. DAG analysis performed using DAGitty v.2.3 online platform. **Figure S2.** Correlations (Pearson’s) for study variables, including LINE-1 and Alu methylation. **Table S1.** Summary statistics (mean (95%CI)) for cell type concentration overall and stratified by SES indicators. **Table S2.** Results from linear regression mixed effects (LMER) models of crude and adjusted associations between maternal, household, and neighborhood indicators of SES and diet quality index and Alu DNA methylation. (DOC 665 kb)

## Abbreviations

BMI: Body mass index
CHAMACOS: Center for Health Assessment of Mothers and Children of Salinas
DAG: Directed acyclic graph
DQI-P: Diet Quality Index for Pregnancy
FFQ: Food-frequency questionnaire
LINEs: Long interspersed nuclear elements
MBzP: Monobenzyl phthalate
SES: Socioeconomic status
SINEs: Short interspersed nuclear elements
