# Supplementary material for: DNA methylation and socioeconomic status in a Mexican-American birth cohort
Source: Clin Epigenetics. 2018 May 8;10:61. doi: 10.1186/s13148-018-0494-z (PMC5941629; doi:10.1186/s13148-018-0494-z)
Supplement: Supplementary file 1 — Figure S1. Minimal sufficient adjustment for measuring the direct effect of SES on LINE-1 DNA methylation: diet quality index, maternal urinary phthalate concentration, maternal age, maternal smoking during pregnancy, and the number of years living in the U.S. DAG analysis performed using DAGitty v.2.3 online platform. Figure S2. Correlations (Pearson’s) for study variables, including LINE-1 and Alu methylation. Table S1. Summary statistics (mean (95%CI)) for cell type concentration overall and stratified by SES indicators. Table S2. Results from linear regression mixed effects (LMER) models of crude and adjusted associations between maternal, household, and neighborhood indicators of SES and diet quality index and Alu DNA methylation. (DOC 665 kb) [file 13148_2018_494_MOESM1_ESM.doc]

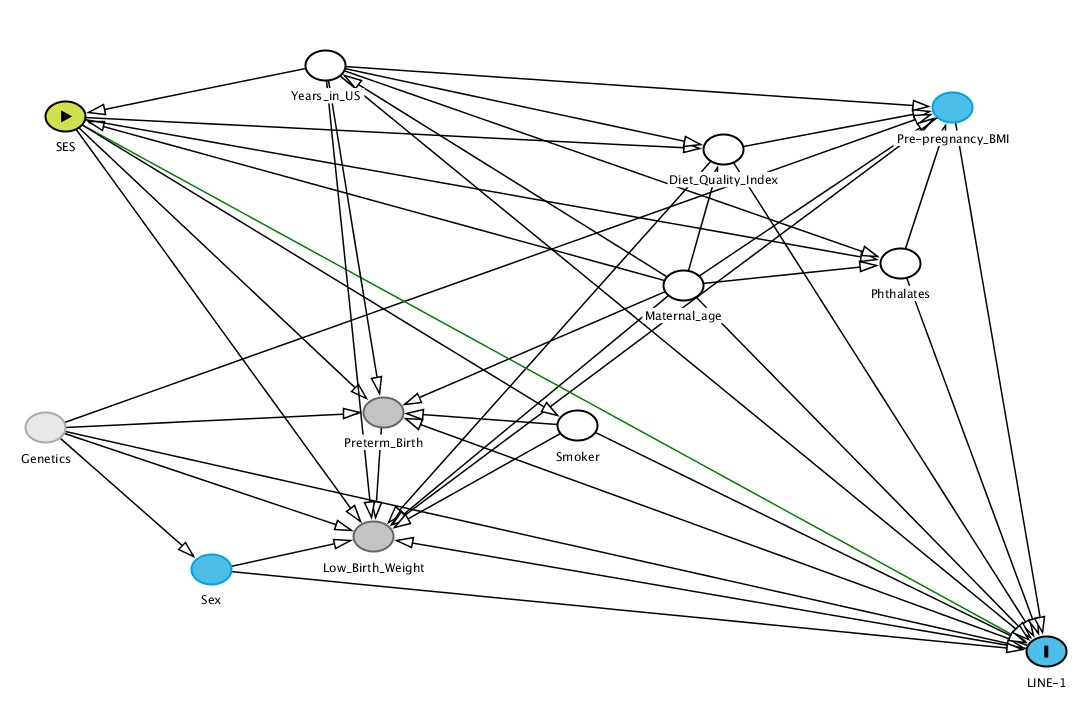

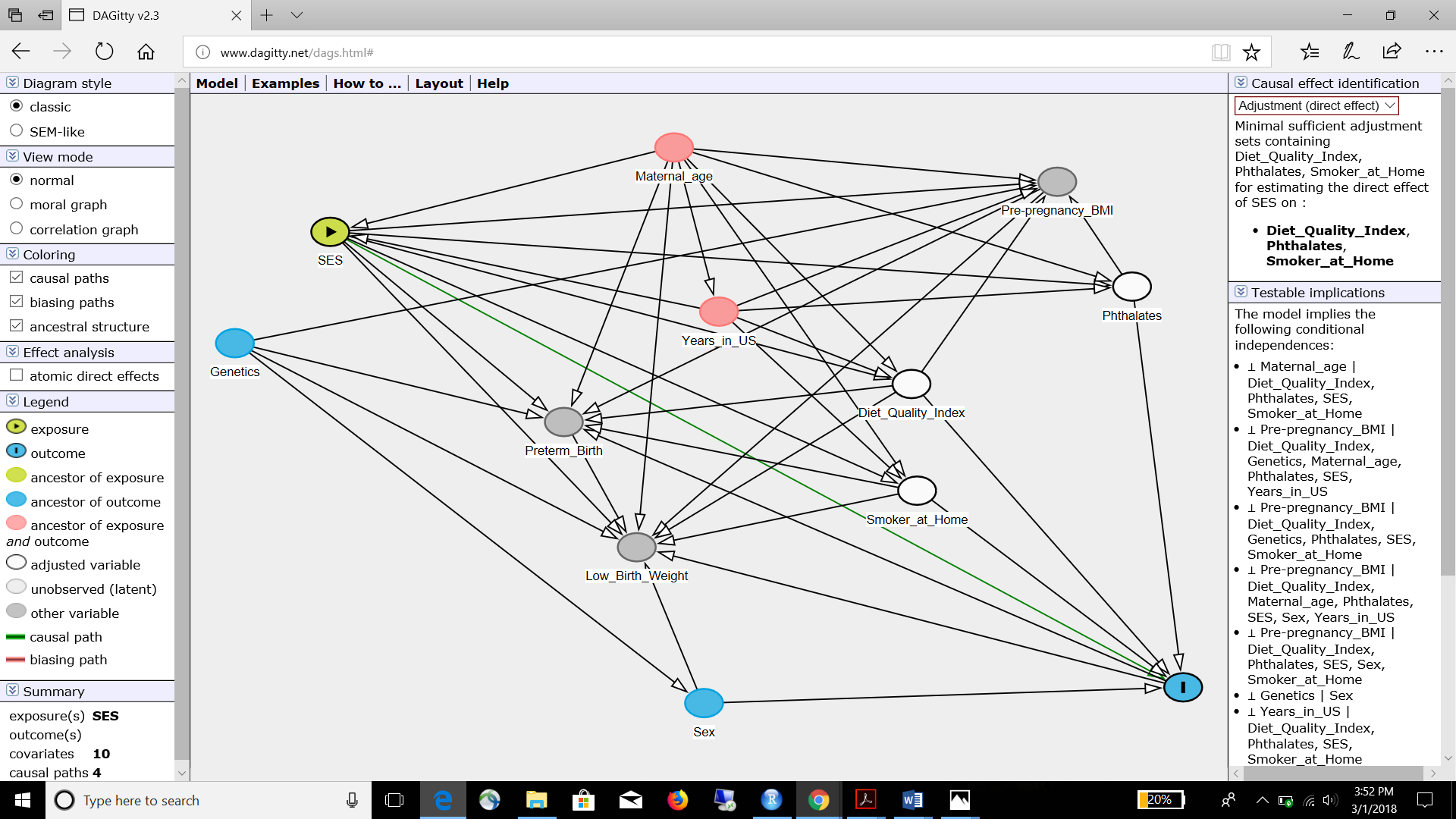


Figure S1. Minimal sufficient adjustment for measuring the direct effect of SES on LINE-1 DNA methylation: diet quality index, maternal urinary phthalate concentration, maternal age, maternal smoking during pregnancy, and the number of years living in the U.S. DAG analysis performed using DAGitty v.2.3 online platform.


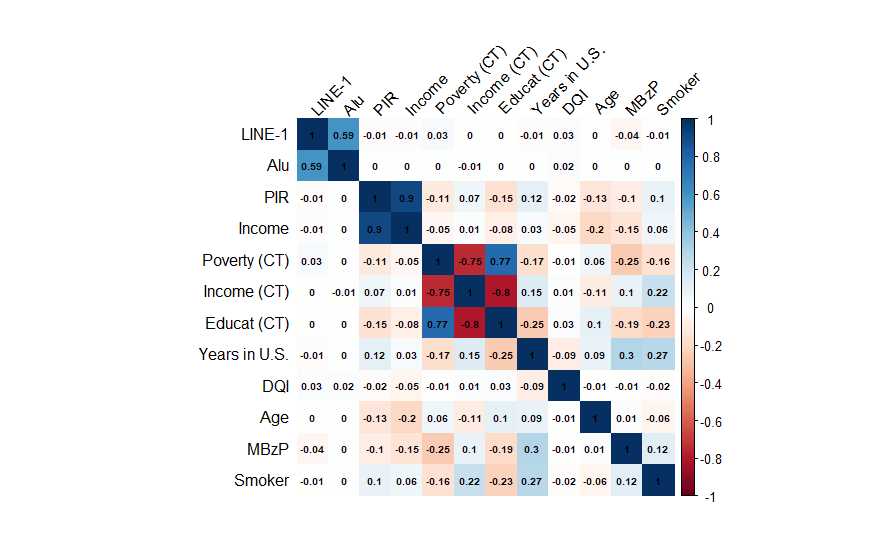


Figure S2. Correlations (Pearson’s) for study variables, including LINE-1 and Alu methylation.

| Table S1. Summary statistics (mean [95%CI]) for cell-type concentration overall and stratified by SES indicators^a^. | | | | | | | |
| --- | --- | --- | --- | --- | --- | --- | --- |
|  | **CD8 T-cells** | **CD4 T-cells** | **Natural killer T-cells** | **B lymphocytes** | **Monocytes** | **Granulocytes** | **Nucleated RBCs** |
| Overall | 0.101  (0.096, 0.106) | 0.107  (0.100, 0.114) | 0.026  (0.023, 0.028) | 0.081  (0.769, 0.085) | 0.090  (0.087, 0.093) | 0.460  (0.448, 0.472) | 0.171  (0.158, 0.183) |
| **Maternal SES Indicators** | | | | | | | |
| Educational Attainment |  |  |  |  |  |  |  |
| <6^th^ Grade | 0.102  (0.094, 0.110) | 0.104  (0.092, 0.116) | 0.025  (0.021, 0.028) | 0.077  (0.072, 0.083) | 0.087  (0.082, 0.091) | 0.465  (0.447, 0.483) | 0.175  (0.155, 0.195) |
| 7^th^ – 12^th^ Grade | 0.100  (0.092, 0.107) | 0.110  (0.098, 0.122) | 0.026  (0.021, 0.031) | 0.080  (0.074, 0.086) | 0.093  (0.088, 0.098) | 0.459  (0.440, 0.478) | 0.168  (0.147, 0.188) |
| ≥ High School | 0.102  (0.089, 0.114) | 0.107  (0.090, 0.124) | 0.028  (0.022, 0.034) | 0.088  (0.072, 0.099) | 0.092  (0.086, 0.099) | 0.451  (0.419, 0.483) | 0.166  (0.139, 0.194) |
| **Household SES Indicators** | | | | | | | |
| Monthly Income Quartiles |  |  |  |  |  |  |  |
| 1^st^ Quartile ($37 - $225) | 0.109  (0.094, 0.125) | 0.089  (0.065, 0.112) | 0.028  (0.020, 0.036) | 0.080  (0.067, 0.094) | **0.080**  **(0.071, 0.089)** | 0.428  (0.383, 0.472) | **0.218**  **(0.174, 0.262)** |
| 2^nd^ Quartile ($281 - $375) | 0.093  (0.082, 0.104) | 0.100  (0.086, 0.114) | 0.024  (0.018, 0.030) | 0.077  (0.070, 0.084) | **0.092**  **(0.086, 0.098)** | 0.477  (0.452, 0.503) | **0.171**  **(0.143, 0.200)** |
| 3^rd^ Quartile ($438 - $563) | 0.102  (0.094, 0.109) | 0.117  (0.105, 0.128) | 0.023  (0.019, 0.027) | 0.081  (0.076, 0.087) | **0.094**  **(0.089, 0.099)** | 0.466  (0.450, 0.483) | **0.156**  **(0.138, 0.174)** |
| 4^th^ Quartile ($583 - $1750) | 0.105  (0.092, 0.118) | 0.106  (0.085, 0.128) | 0.029  (0.023, 0.035) | 0.085  (0.073, 0.097) | **0.087**  **(0.081, 0.092)** | 0.449  (0.416, 0.482) | **0.170**  **(0.141, 0.199)** |
| Poverty Income Ratio Quartiles |  |  |  |  |  |  |  |
| 1^st^ Quartile (0.13- 0.65) | 0.102  (0.089, 0.114) | 0.088  (0.068, 0.108) | 0.026  (0.019, 0.032) | 0.079  (0.068, 0.090) | 0.083  (0.076, 0.091) | 0.440  (0.404, 0.475) | **0.214**  **(0.177, 0.252)** |
| 2^nd^ Quartile (0.71- 0.98) | 0.097  (0.086, 0.107) | 0.103  (0.091, 0.116) | 0.026  (0.020, 0.031) | 0.080  (0.073, 0.086) | 0.091  (0.086, 0.097) | 0.476  (0.453, 0.499) | **0.163**  **(0.140, 0.187)** |
| 3^rd^ Quartile (1.01- 1.21) | 0.102  (0.094, 0.109) | 0.116  (0.103, 0.129) | 0.021  (0.016, 0.026) | 0.080  (0.074, 0.086) | 0.094  (0.089, 0.099) | 0.465  (0.447, 0.483) | **0.159**  **(0.137, 0.181)** |
| 4^th^ Quartile (1.30 – 2.40) | 0.107  (0.092, 0.122) | 0.114  (0.092, 0.137) | 0.032  (0.025, 0.038) | 0.087  (0.075, 0.099) | 0.087  (0.080, 0.094) | 0.445  (0.410, 0.481) | **0.160**  **(0.135, 0.186)** |
| **Neighborhood SES Indicators** | | | | | | | |
| Median Household Income Quartiles |  |  |  |  |  |  |  |
| 1^st^ Quartile ($24896 – $31910) | 0.107  (0.097, 0.117) | 0.110  (0.097, 0.124) | 0.029  (0.023, 0.035) | 0.081  (0.073, 0.089) | 0.090  (0.083, 0.096) | 0.460  (0.434, 0.485) | 0.159  (0.141, 0.178) |
| 2^nd^ Quartile ($31989 – $34593) | 0.098  (0.091, 0.105) | 0.107  (0.094, 0.119) | 0.024  (0.019, 0.028) | 0.081  (0.075, 0.081) | 0.090  (0.086, 0.095) | 0.453  (0.435, 0.472) | 0.182  (0.159, 0.205) |
| 3^rd^ Quartile ($34848 – $40856) | 0.096  (0.081, 0.111) | 10.106  (0.088, 0.123) | 0.026  (0.021, 0.031) | 0.076  (0.067, 0.076) | 0.092  (0.085, 0.100) | 0.479  (0.445, 0.512) | 0.158  (0.127, 0.189) |
| 4^th^ Quartile ($41354 – $77272) | 0.104  (0.091, 0.117) | 0.104  (0.085, 0.123) | 0.026  (0.020, 0.032) | 0.085  (0.075, 0.096) | 0.088  (0.081, 0.096) | 0.455  (0.428, 0.483) | 0.175  (0.148, 0.201) |
| Percent Below Poverty Quartiles |  |  |  |  |  |  |  |
| 1^st^ Quartile (2.8 – 18.2) | 0.106  (0.096, 0.115) | 0.108  (0.093, 0.123) | 0.027  (0.022, 0.034) | 0.081  (0.073, 0.089) | **0.087**  **(0.080, 0.093)** | 0.463  (0.438, 0.489) | 0.167  (0.147, 0.188) |
| 2^nd^ Quartile (19.0 – 22.3) | 0.101  (0.089, 0.112) | 0.107  (0.091, 0.123) | 0.028  (0.023, 0.033) | 0.084  (0.075, 0.092) | **0.092**  **(0.085, 0.098)** | 0.452  (0.427, 0.476) | 0.171  (0.148, 0.194) |
| 3^rd^ Quartile (23.5 – 27.5) | 0.101  (0.093, 0.109) | 0.106  (0.095, 0.118) | 0.024  (0.020, 0.028) | 0.079  (0.073, 0.085) | **0.088**  **(0.084, 0.092)** | 0.457  (0.438, 0.477) | 0.179  (0.155, 0.202) |
| 4^th^ Quartile (27.7 – 34.2) | 0.0921  (0.080, 0.104) | 0.107  (0.078, 0.136) | 0.024  (0.015, 0.033) | 0.079  (0.068, 0.090) | **0.102**  **(0.093, 0.111)** | 0.482  (0.443, 0.521) | 0.144  (0.113, 0.175) |
| Percent without a Highschool Education Quartiles |  |  |  |  |  |  |  |
| 1^st^ Quartile (13.7 – 50.1) | 0.105  (0.095, 0.115) | 0.111  (0.096, 0.125) | 0.028  (0.022, 0.031) | 0.081  (0.074, 0.089) | **0.087**  **(0.081, 0.094)** | 0.453  (0.427, 0.480) | 0.170  (0.148, 0.170) |
| 2^nd^ Quartile (51.4 – 71.4) | 0.097  (0.088, 0.107) | 0.107  (0.090, 0.123) | 0.025  (0.020, 0.031) | 0.082  (0.074, 0.091) | **0.095**  **(083, 0.101)** | 0.466  (0.444, 0.488) | 0.162  (0.141, 0.183) |
| 3^rd^ Quartile (72.5 – 75.3) | 0.102  (0.090, 0.113) | 0.106  (0.093, 0.120) | 0.027  (0.022, 0.031) | 0.083  (0.074, 0.091) | **0.090**  **(0.084, 0.095)** | 0.466  (0.442, 0.490) | 0.162  (0.136, 0.189) |
| 4^th^ Quartile (78.7 – 87.0) | 0.100  (0.091, 0.109) | 0.104  (0.088, 0.120) | 0.023  (0.018, 0.028) | 0.077  (0.069, 0.084) | **0.090**  **(0.084, 0.095)** | 0.455  (0.429, 0.481) | 0.188  (0.158, 0.217) |
| ^a^Bold print indicates statistically significant (p<0.05) difference in mean cell levels between SES categories using ANOVA. | | | | | | | |

|  |  | |  | |
| --- | --- | --- | --- | --- |
| Table S2. Results from linear regression mixed-effects (LMER) models of crude and adjusted associations between maternal, household, and neighborhood indicators of SES and Diet Quality Index and Alu DNA methylation. | | | | |
| Social Adversity Indicators | Model 1^a^ | | Model 2^b^ | |
|  | β (95% CI) | p-value | aβ (95% CI) | p-value |
| Household Income |  |  |  |  |
| Lowest Income Quartile | -0.15 (-0.91, 0.60) | 0.69 | -0.12 (-0.92, 0.68) | 0.77 |
| Second Income Quartile | -0.04 (-0.72, 0.65) | 0.91 | -0.02 (-0.74, 0.70) | 0.96 |
| Third Income Quartile | -0.06 (-0.83, 0.72) | 0.89 | 0.01 (-0.78, 0.80) | 0.97 |
| Highest Income Quartile | Reference |  |  |  |
| Household Poverty Income Ratio |  |  |  |  |
| Lowest Poverty Ratio Quartile | -0.11 (-0.77, 0.55) | 0.75 | -0.11 (-0.80, 0.58) | 0.75 |
| Second Poverty Ratio Quartile | -0.07 (-0.70, 0.57) | 0.83 | -0.09 (-0.75, 0.57) | 0.78 |
| Third Poverty Ratio Quartile | -0.04 (-0.78, 0.70) | 0.92 | 0.01 (-0.75, 0.77) | 0.98 |
| Highest Poverty Ratio Quartile | Reference |  | Reference |  |
| Maternal Education |  |  |  |  |
| <= 6th grade | -0.18 (-0.76, 0.40) | 0.54 | -0.18 (-0.80, 0.43) | 0.56 |
| 7-12th grade | 0.02 (-0.57, 0.61) | 0.94 | -0.06 (-0.68, 0.56) | 0.85 |
| >=High School | Reference |  | Reference |  |
| % Households Below Poverty (CT) |  |  |  |  |
| Lowest Poverty Quartile | Reference |  | Reference |  |
| Second Poverty Quartile | -0.06 (-0.69, 0.56) | 0.84 | -0.08 (-0.73, 0.56) | 0.80 |
| Third Poverty Quartile | -0.04 (-0.60, 0.53) | 0.89 | -0.06 (-0.67, 0.54) | 0.84 |
| Highest Poverty Quartile | 0.04 (-0.78, 0.86) | 0.92 | 0.03 (-0.84, 0.91) | 0.94 |
| Median Household Income (CT) |  |  |  |  |
| Lowest Income Quartile | -0.04 (-0.59, 0.51) | 0.89 | -0.05 (-0.64, 0.53) | 0.86 |
| Second Income Quartile | -0.02 (-0.69, 0.66) | 0.96 | 0.04 (-0.68, 0.76) | 0.92 |
| Third Income Quartile | -0.10 (-0.78, 0.58) | 0.78 | -0.14 (-0.83, 0.56) | 0.70 |
| Highest Income Quartile | Reference |  | Reference |  |
| % No High School Education (CT) |  |  |  |  |
| Lowest Education Quartile | Reference |  | Reference |  |
| Second Education Quartile | -0.02 (-0.65, 0.61) | 0.95 | -0.07 (-0.72, 0.59) | 0.84 |
| Third Education Quartile | 0.13 (-0.47, 0.73) | 0.68 | 0.15 (-0.49, 0.78) | 0.65 |
| Highest Education Quartile | -0.05 (-0.66, 0.55) | 0.86 | -0.10 (-0.75, 0.55) | 0.77 |
| Diet Quality Index | 0.01 (-0.01, 0.04) | 0.29 | 0.01 (-0.01, 0.03) | 0.40 |
| ^a^Model 1: Random effect for position and individual only  ^b^Model 2: Random effect for position and individual, maternal smoking during pregnancy, maternal age, diet quality during pregnancy, years living in the U.S. for the mother, and prenatal MBzP exposure.  ^c^Model 3: Random effect for position and individual, maternal smoking during pregnancy, maternal age, diet quality during pregnancy, years living in the U.S. for the mother, prenatal MBzP exposure, and cell estimate proportions.  ^d^Neighborhood poverty included as a covariate due to evidence of confounding by neighborhood poverty. Diet quality index was Z-standardized so that continuous variables were on similar scales. | | | | |
